# Gender differences in association between metabolic syndrome and carotid intima media thickness

**DOI:** 10.1186/2251-6581-11-13

**Published:** 2012-09-07

**Authors:** Ozra Tabatabaei-Malazy, Hossein Fakhrzadeh, Farshad Sharifi, Mojde Mirarefin, Zohre Badamchizadeh, Bagher Larijani

**Affiliations:** 1 Endocrinology & Metabolism Research Center, Tehran University of Medical Sciences, Fifth floor, Dr. Shariati Hospital, North Kargar Ave, Tehran, 14114, Iran

**Keywords:** Metabolic syndrome, Cardiovascular disease, Carotid intima-media thickness, Sex

## Abstract

**Background:**

Metabolic syndrome (Mets) is a cluster of cardiovascular risk factors which can predicts cardiovascular disease (CVD). Carotid intima-media thickness (CIMT) is known as a surrogate measure of subclinical atherosclerosis and predictor of CVD. Although, it has shown the association between Mets and CIMT, this relation regarding sex differences is limited. We aimed to find out whether gender differences in this association.

**Methods:**

In this cross-sectional study, we recorded height, weight, waist circumference (WC), blood pressure, and lipid profiles. We used Mets; defined based on NCEP ATP III definition, and traditional cardiovascular risk factors; age, body mass index (BMI), WC, hyperlipidemia, and hypertension, in multivariate regression models which including;. The CIMT measurement < 0.73 or ≥0.73 mm was considered as low- or high risk to CVD.

**Results:**

Overall, 150 subjects were enrolled to study that their ages were 36-75 years. The 47.3% of them (71 subjects) had Mets. CIMT was increased in Mets group compared non-Mets group (P = 0.001). In logistic regression analysis, a significant association was found between Mets and CIMT in women, but not in men (p = 0.002, and p = 0.364, respectively). After adjustment to age, WC, BMI, hypertension and hyperlipidemia, this association was significant just in women (p = 0.011) independent of WC, BMI, hyperlipidemia and hypertension.

**Conclusion:**

Our data showed that MetS is a stronger risk factor for subclinical atherosclerosis in women than in men. So, we suggest the assessment of CIMT along with definition Mets in middle-aged women could be lead to earlier detection of at risk individuals to CVD.

## Introduction

Metabolic syndrome (MetS) is defined as a cluster of cardiovascular risk factors including central obesity, hypertension, dyslipidemia, and glucose intolerance [1]. It has been shown that MetS is a predictor of type 2 diabetes mellitus (T2DM) [2] and cardiovascular disease (CVD) [3]. MetS is associated with an approximately two fold increase in CVD [4]. The Third National Health and Nutrition Examination Survey (TNHNES) reported that the prevalence of MetS was 24% in adults older than 20 years and 42% in individuals aged 70 years or older [5]. Its age-adjusted prevalence among adults aged 25- 64 years who participated in the MONICA WHO Study in Iran was estimated at 27.5% [6]. The prevalence was significantly higher in women than in men (35.9% vs. 20.3%).

Carotid artery intima-media thickness (CIMT) is a non-invasive surrogate marker of atherosclerosis [7]. Its progression is influenced by conventional CVD risk factors [8]. Measurement of CIMT can directly predict the risk of future cardiovascular events [8]. Increasing age, male gender, hypertension, obesity, and T2DM or glucose intolerance are associated with accelerated atherosclerosis in the carotid arteries [8].

Several studies have reported an association between MetS and increased CIMT [9-13]. It has been suggested that MetS is a stronger risk factor for atherosclerosis in women than in men [14,15], although there are other reports which do not confirm this finding [12,16]. To our knowledge possible sex differences regarding this issue have not been studied in Iran and we aimed to investigate this subject in a cross-sectional survey.

## Methods

### Study population

We used data from a subgroup of participants who were enrolled in the Rapid Atherosclerosis Prevention In Diabetes (RAPID) study. The RAPID study is an ongoing prospective single-center cohort study in subjects aged ≥30 years and without any clinical evidence of coronary artery disease (Minnesota codes 1.2.1, 1.2.4, 1.2.5, and 1.2.7) at the time of the investigation. This study was started in September 2010 at Dr. Shariati Hospital/Tehran University of Medical Sciences (TUMS), for early detection of atherosclerosis in T2DM patients. Written informed consent was obtained from all participants. The study was approved by the ethics committee of the TUMS. In the present study we compared carotid stiffness between subjects with and without MetS. Participants with clinical evidence of coronary artery disease; angina, positive ST elevation in ECG, or CVD end-points such as myocardial infarction (MI) or stroke were excluded. We recorded characteristics of 150 participants of whom 71 (47.3%) suffered from MetS. Mean age of the participants was 49.8 ± 7.5 years (range: 39 years).

### Definitions

Mets was diagnosed according to NCEP ATP III guidelines [1]. According to NCEP ATP III definition, MetS was confirmed if at least three of the following criteria were present: waist circumference (WC) ≥102 cm (in men) or ≥88 cm (in women), triglyceride ≥150 mg/dl or a history of previous treatment for dyslipidemia, HDL cholesterol ≤40 mg/dl (in men) or ≤50 mg/dl (in women), blood pressure ≥130/85 mmHg or those who had been treated for hypertension, and fasting blood sugar (FBS) ≥ 110 mg/dl or a history of previous treatment for diabetes [1].

CVD is a group of heart and blood vessel disorders that include coronary heart disease, cerebrovascular disease, peripheral arterial disease, and congenital heart disease, etc [17].

Heart attacks and strokes are usually acute events caused by a blockage that prevents blood from flowing to the heart or brain. The most common reason is a build-up of fatty deposits on the inner walls of the blood vessels [17].

The term MI reflects cell death of cardiac myocytes which is caused by ischemia. In other words it is the result of a perfusion imbalance between supply and demand. MI should be diagnosed by symptoms, ECG abnormalities and enzymes, specific serological biomarkers and imaging [18].

Angina (or angina pectoris) is a symptom of coronary artery disease caused by reduced blood flow to the heart muscle leading to chest pain [19].

### Conventional cardiovascular risk factors

Hypertension, WC, and hyperlipidemia were defined according to NCEP ATP III guidelines [1]. In each subject (at standing position), WC was measured with a tape in centimeters as the widest value between the margin of lower limb and iliac crest. Blood pressure was measured twice (5 minutes interval) using a standard calibrated mercury sphygmomanometer on both right and left hands after the participants had been sitting for at least 10 minutes. The highest blood pressure of two sides was considered as blood pressure. Lipid profiles (total cholesterol >200 mg/dl, low density lipoprotein >100 mg/dl, and triglyceride >150 mg/dl) were considered as hyperlipidemia according to NCEP ATP III guidelines [1]. Diabetes was defined when fasting blood sugar (FBS) was ≥ 110 mg/dl or there was a history of previous treatment for diabetes. Height was measured in standing position and weight was measured twice with at least clothes and without shoes for calculating BMI by this formula: weight (kg)/height^2^ (m^2^).

### Assessment of CIMT

We used high-resolution B-mode carotid ultrasound scanner equipped with a linear 12 MHz transducer (My Lab 70 X Vision. Biosound Esaote, USA) for examination of the right and left carotid arteries. The examinations were performed by an expert in 12 locations as the following: Right anterior and posterior internal carotid arteries, Left anterior and posterior internal carotid arteries, Right anterior and posterior carotid artery bifurcation, Left anterior and posterior carotid artery bifurcation, Right anterior and posterior common carotid artery, and Left anterior and posterior common carotid artery [20]. A segment of the artery which was most clearly visible to the examiner was magnified to identify a distinct lumen-intima and media-adventitia interface. CIMT was defined as the distance between the leading edge of the lumen-intima interface and the leading edge of the media-adventitia interface. Maximum thickness was measured semi-automatically off-line with artery measurement system software (Vascular tools 5, Medical Imaging Applications LLC, USA). The cut-off point of ≥0.73 mm was considered as high risk for development of atherosclerotic vascular disease [21].

### Laboratory tests

Venous blood samples were drawn from the ante-cubital vein after an overnight fast to measure fasting blood sugar (FBS), total cholesterol, triglyceride, and high-density lipoprotein (HDL). These biochemical tests were determined enzymatically with Pars- Azmon kit/Iran. Low-density lipoprotein (LDL) cholesterol was calculated with the Friedewald formula if triglyceride level was met <400 mg/dl [22]; it was measured directly in participants whose triglyceride was ≥400 mg/dl.

### Statistical analyses

The normality of distribution of data was evaluated by Kolmogrov-Smirnov test. All obtained values were expressed as mean ± standard deviation (SD). Paired *T*-Test was applied for variables with normal distribution, and Wilcoxon and Mann–Whitney nonparametric tests for other variables. Univariate and multivariate logistic regression models were performed to examine association between Mets and conventional risk factors of CVD with CIMT separately in men and women. Statistical analyses were performed using SPSS, version 15.0 and P value ≤0.05 was considered as statistically significant.

## Results

Majority of the participants [87 (58%)] were females. Prevalence of hyperlipidemia (hypertriglyceridemia + hypercholesterolemia) and hypertension was 126 (84%) and 66 (44%), respectively. Within participants with MetS, the percent of MetS components was as follow: 10% without component, 22.7% one component, 20% two components, 20.7% three components, 21.3% four components, and 5.3% five components. Table 1 shows the baseline characteristics of participants with and without MetS. We found in regression analysis that is an interaction with sex in the association between MetS and CIMT. The statistical difference (p) among men was 0.053 and among women was <0.001. Results of univariate and multivariate regression models separately in men and women are shown in Table 2, and Table 3, respectively.

**Table 1:** Baseline characteristics of 150 participants with and without MetS

**Variable**	**Non-MetS group**	**MetS group**	**P Value**
	**n = 79**	**n = 71**	
	**(mean ± SD)**	**(mean ± SD)**	
**age (yr)**	49.2 ± 8.2	50.5 ± 6.7	0.32
**Sex (M/F) (n (%))**	24(16%)/55(36.7%)	39(26%)/32(21.3%)	0.003*
**BMI (kg/m**^**2**^**)**	27.8 ± 4.6	29.1 ± 3.8	0.03*†
**WC (cm)**	87.4 ± 10.7	97.1 ± 8.2	<0.001*†
**SBP (mmHg)**	118 ± 14	135 ± 15	<0.001*
**DBP (mmHg)**	74 ± 9	82 ± 11	<0.001*
**FBS (mg/dl)**	89 ± 9	100 ± 12	<0.001*†
**TChol (mg/dl)**	196 ± 35	205 ± 33	0.10
**TG (mg/dl)**	128 ± 57	197 ± 85	<0.001*†
**LDL (mg/dl)**	110 ± 23	118 ± 22	0.01*†
**HDL (mg/dl)**	50 ± 10	42 ± 9	<0.001*†
**CIMT (mm)**	0.60 ± 0.11	0.69 ± 0.16	0.001*†

**Legend: *M/F*: **Male/Female, ***BMI*: **Body Mass Index, ***WC*: **Waist Circumference, ***SBP*: **Systolic Blood Pressure, ***DBP*: **Diastolic Blood Pressure, ***FBS*: **Fasting Blood Sugar, ***TChol*: **Total Cholesterol, ***TG*: **Triglyceride, ***LDL*: **Low Density lipoprotein, ***HDL*: **High Density Lipoprotein, ***CIMT*: **Carotid artery Intima Media Thickness.

*P ≤ 0.05 was considered statistically significant.

^†^Analytic test was Mann–Whitney Test. In others the analytic test was *T*-Test.

**Table 2:** Association between CIMT and MetS in men by univariate and multivariate regression models

**Variable**	**Univariate**		**Multivariate**	
	**Odds ratio**	**P Value**	**Odds ratio**	**P Value**
	**(CI 95%)**		**(CI 95%)**	
**MetS (yes/no)**	1.833	0.364	0.319	0.462
	(0.496-6.778)		(0.015-6.707)	
**Age (yr)**	1.112	0.015*	1.223	0.005*
	(1.021-1.211)		(1.062-1.408)	
**BMI (kg/m**^**2**^**)**	1.077	0.339	1.182	0.364
	(0.925-1.255)		(0.824-1.697)	
**WC (cm)**	1.018	0.537	0.941	0.399
	(0.962-1.078)		(0.818-1.083)	
**Diabetes (yes/no)**	1.312	0.656	1.629	0.604
	(0.397-4.338)		(0.258-10.288)	
**Hyperlipidemia (yes/no)**	6.000	0.101	49.363	0.045*
	(0.707-50.920)		(1.100-2215.205)	
**Hypertension (yes/no)**	1.500	0.509	5.263	0.114
	(0.450-5.003)		(0.672-41.247)	

**Legend: *MetS*: **Metabolic syndrome, ***BMI*: **Body Mass Index, ***WC*: **Waist Circumference, ***CI 95%*: **Confidence Interval 95%.

*P ≤ 0.05 was considered statistically significant in Binary regression models.

**Table 3:** Association between CIMT and MetS in women by univariate and multivariate regression models

**Variable**	**Univariate**		**Multivariate**	
	**Odds ratio**	**P Value**	**Odds ratio**	**P Value**
	**(CI 95%)**		**(CI 95%)**	
**MetS (yes/no)**	6.750	0.002*	14.223	0.011*
	(2.055-22.167)		(1.828-110.649)	
**Age (yr)**	1.115	0.012*	1.208	0.014*
	(1.024-1.213)		(1.039-1.404)	
**BMI (kg/m**^**2**^**)**	0.951	0.426	0.792	0.106
	(0.839-1.077)		(0.597-1.051)	
**WC (cm)**	1.021	0.444	0.970	0.649
	(0.968-1.076)		(0.849-1.107)	
**Diabetes (yes/no)**	1.035	0.154	0.811	0.820
	(0.987-1.086)		(0.133-4.941)	
**Hyperlipidemia (yes/no)**	2.415	0.422	0.666	0.746
	(0.281-20.790)		(0.057-7.810)	
**Hypertension (yes/no)**	3.697	0.022*	1.769	0.475
	(1.211-11.291)		(0.370-8.463)	

**Legend: *MetS*: **Metabolic syndrome, ***BMI*: **Body Mass Index, ***WC*: **Waist Circumference, ***CI 95%*: **Confidence Interval 95%.

*P ≤ 0.05 was considered statistically significant in Binary regression models.

## Discussion

We found that CIMT in asymptomatic middle-aged adults with MetS was higher than those without MetS (p = 0.001). Furthermore, regression analyses for conventional risk factors both unadjusted and adjusted, indicated that this association was significant only in women (p = 0.002, p = 0.011, respectively). These findings highlight the importance of an increased burden of subclinical atherosclerosis in middle-aged women with MetS and suggest the presence of an increased risk of future CVD in this group. It has been shown that patients with MetS are at increased risk for development of vascular abnormalities ranged from endothelial dysfunction, followed by artery stiffness to evident atherosclerosis [23]. A meta-analysis showed that subjects with MetS have 61% higher risk of CVD than those without MetS [24].

Several cross-sectional studies have shown a significant association between CIMT and MetS [11-13,25]. Hulthe et al [10] reported an association between MetS with accelerated atherosclerosis in asymptomatic middle-aged adults. The Botnia Study demonstrated that middle-aged adults with MetS have an approximately three-fold increased risk of incident CVD [26]. The Bogalusa Heart Study [27] reported that MetS, defined by either NCEP ATP III or WHO criteria, was associated with increased CIMT.

We chose NCEP ATP III criteria for definition of MetS due to its easy applicability to clinical practice and epidemiological studies. In addition, in this method we do not require insulin measurement, albumin assessment in urine or oral glucose tolerance test (OGTT) as needed for definition MetS according to WHO [28]. So, the NCEP ATP III criteria may be less sensitive than the WHO criteria in predicting T2DM [29]. In contrast, it was found that subjects with MetS according to NCEP ATP III criteria were less insulin resistant and at higher risk for future CVD than subjects with MetS by WHO definition [30]. Because of differing definitions of MetS in various articles, comparison with previous studies is difficult. The Atherosclerosis and Insulin Resistance Study showed that middle-aged white men with MetS according to WHO criteria had increased CIMT [10], whereas the Brunek Study demonstrated that CIMT was significantly higher in middle-aged and elderly adults with MetS according to NCEP ATP III and modified WHO definitions [3]. By other diagnostic criteria including International Diabetes Federation (IDF) and American Heart Association/National Heart, Lung, and Blood Institute (AHA/NHLBI), the sex differences in association between CIMT and MetS were not homogenous [13]. However, irrespective of definition, CIMT was significantly higher in both men and women with MetS than those without MetS [13].

The gender difference association between MetS and CIMT in our study was in line with findings of Iglseder et al [14] who also found this significant association only in women. This finding may be due to higher levels of C - reactive protein (CRP) and inflammatory markers in pre-menopausal women [31]. Higher levels of fasting leptin and lower insulin sensitivity in pre-menopausal women may also play a role in this context [32].

On the other hand, the results of study by Skilton et al [13] showed no sex differences in the association between MetS and CIMT by NCEP ATP III criteria, although this relation was apparent when they used other criteria for definition of MetS [13]. This finding stipulates that female protection against atherosclerosis is lost in the presence of MetS. This suggestion is supported by identification of sex-specific and sex-independent quantitative trait loci for MetS components in animal studies [33]. It is also supported by observation of the influence of sex on several components of MetS among male and female twins [34].

When we assessed the effect of MetS on CIMT after adjusting for traditional cardiovascular risk factors in the multivariate regression model, MetS was a significant independent predictor of CVD only in women (p = 0.011). We found some differences between both sexes in traditional risk factors affecting CIMT. As expected, age was the strongest determinant of CIMT in both sexes. This finding is in line with previous studies [11,35]. Hyperlipidemia was another determinant which was effective only in men with. The Atherosclerosis Risk in Communities (ARIC) study [7] demonstrated an association between CIMT and incidence of MI even after adjusting for age, race, diabetes, cholesterol, hypertension and smoking in a large population study of middle-aged adults. In addition, the Cardiovascular Health Study showed a significant association between CIMT and risk for CVD after adjusting for traditional risk factors [36]. In the Muscatine Study, multivariate analysis showed that CIMT was associated with systolic blood pressure, increasing age, and LDL in women and with smoking in men [37]. In the Health 2000 Survey [9] it was found that BMI, WC, LDL, total cholesterol and diastolic blood pressure had statistically significant univariate correlation with CIMT in women but not in men. However, our results suggested that MetS screening in hyperlipidemic men provides more benefit than women to identify subjects at risk for CVD. This finding may be related to gender-specific differences in the association between LDL cholesterol and atherosclerosis [38]. However, we cannot address any cause and effect relationship regarding the effect of hyperlipidemia on CIMT due to cross-sectional nature of our study. Furthermore, most subjects were being under treatment with several types of medications for hyperlipidemia. These medications have direct effects on vascular function to halt the progression or even reduce CIMT [39].

Our study had several limitations. Because our study was carried out in a small population, the observed effects may not be applicable to the general population. In some studies it has been observed that CIMT increases with increasing number of MetS components [40-42]. This supports the hypothesis that declares clustering of MetS components has an additive effect on progression of CIMT [41]. However, we were not able to investigate this hypothesis in our study due to limitation in sample size. Thus, prospective studies are required to determine the ability of each component of MetS to predict the occurrence of cardiovascular events in women.

## Conclusion

It seems that MetS provides useful information of the patient’s cardiovascular risk in addition to the traditional risk factors. The burden of subclinical atherosclerosis increases in middle-aged with MetS. In addition, increasing age and presence hyperlipidemia are strong predictors of increased CIMT. We suggest the assessment of CIMT along with identification of individuals with MetS to provide appropriate intervention for prevention of CVD in middle-aged individuals especially in old women.

## Competing interests

The authors declare that they have no competing interests.

## Authors’ contributions

OT-M conceived of the study, wrote draft the manuscript and performed the statistical analysis. HF was the principle investigator, participated in its design and coordination and helped to draft the manuscript. FS performed the statistical analysis. MM participated in the design of study. ZB collected the data. BL conceived the study. All authors read and approved the final manuscript.

## References

[B1] Expert Panel on Detection, Evaluation, and Treatment of High Blood Cholesterol in Adults Executive Summary of the Third Report of the National Cholesterol Education Program (NCEP) Expert Panel on Detection, Evaluation, and Treatment of High Blood Cholesterol in Adults (Adult Treatment Panel III) JAMA 2001 2486 2497 10.1001/jama.285.19.2486 11368702

[B2] Lorenzo C Okoloise M Williams K Stern MP Haffner SM San Antonio Heart Study The metabolic syndrome as predictor of type 2 diabetes: the San Antonio heart study Diabetes Care 2003 26 3153 3159 10.2337/diacare.26.11.3153 14578254

[B3] Bonora E Kiechl S Willeit J Carotid atherosclerosis and coronary heart disease in the metabolic syndrome prospective data from the Bruneck Study Diabetes Care 2003 26 1251 1257 10.2337/diacare.26.4.1251 12663606

[B4] McNeill AM Rosamond WD Girman CJ The metabolic syndrome and 11-year risk of incident cardiovascular disease in the atherosclerosis risk in communities study Diabetes Care 2005 28 385 390 10.2337/diacare.28.2.385 15677797

[B5] Ford ES Giles WH Dietz WH Prevalence of the metabolic syndrome among US adults: findings from the third National Health and Nutrition Examination Survey JAMA 2002 285 356 359 11790215 10.1001/jama.287.3.356

[B6] Fakhrzadeh H Ebrahimpour P Pourebrahim R Metabolic Syndrome and its Associated Risk Factors in Healthy Adults: A Population-Based Study in Iran Metab Syndr Relat Disord 2006 4 28 34 10.1089/met.2006.4.28 18370767

[B7] Chambless LE Heiss G Folsom AR Association of coronary heart disease incidence with carotid arterial wall thickness and major risk factors: the Atherosclerosis Risk in Communities (ARIC) Study, 1987–1993 Am J Epidemiol 1997 146 483 494 10.1093/oxfordjournals.aje.a009302 9290509

[B8] Sibal L Agarwal SC Home PD Carotid intima-media thickness as a surrogate marker of cardiovascular disease in diabetes Diabetes Metab Syndr Obes 2011 4 23 34 21448319 10.2147/DMSO.S8540 PMC3064409

[B9] Sipilä K Moilanen L Nieminen T Metabolic syndrome and carotid intima media thickness in the Health 2000 Survey Atherosclerosis 2009 204 276 281 10.1016/j.atherosclerosis.2008.08.029 18848324

[B10] Hulthe J Bokemark L Wikstrand J The metabolic syndrome, LDL particle size, and atherosclerosis: the Atherosclerosis and Insulin Resistance (AIR) study Arterioscler Thromb Vasc Biol 2000 20 2140 2147 10.1161/01.ATV.20.9.2140 10978261

[B11] Scuteri A Najjar SS Muller DC Metabolic syndrome amplifies the age associated increases in vascular thickness and stiffness J Am Coll Cardiol 2004 43 1388 1395 10.1016/j.jacc.2003.10.061 15093872

[B12] McNeill AM Rosamond WD Girman CJ Prevalence of coronary heart disease and carotid arterial thickening in patients with the metabolic syndrome (The ARIC Study) Am J Cardiol 2004 94 1249 1254 10.1016/j.amjcard.2004.07.107 15541239

[B13] Skilton MR Moulin P Serusclat A A comparison of the NCEPATPIII, IDF and AHA/NHLBI metabolic syndrome definitions with relation to early carotid atherosclerosis in subjects with hypercholesterolemia or at risk of CVD: evidence for sex-specific differences Atherosclerosis 2007 190 416 422 10.1016/j.atherosclerosis.2006.02.019 16616756

[B14] Iglseder B Cip P Malaimare L The metabolic syndrome is a stronger risk factor for early carotid atherosclerosis in women than in men Stroke 2005 36 1212 1217 10.1161/01.STR.0000166196.31227.91 15890992

[B15] Nishida M Moriyama T Ishii K Effects of IL-6, adiponectin, CRP and metabolic syndrome on subclinical atherosclerosis Clin Chim Acta 2007 384 99 104 10.1016/j.cca.2007.06.009 17618612

[B16] Empana JP Zureik M Gariepy J The metabolic syndrome and the carotid artery structure in non institutionalized elderly subjects: the three-city study Stroke 2007 38 893 899 10.1161/01.STR.0000257983.62530.75 17272758

[B17] Cardiovascular diseases http://www.who.int/who/Europe/cardiovascular/disease. Accessed 2012

[B18] Thygesen K Alpert JS White HD Joint ESC/ACCF/AHA/WHF Task Force for the Redefinition of Myocardial Infarction Universal definition of myocardial infarction Eur Heart J 2007 28 2525 2538 17951287 10.1093/eurheartj/ehm355

[B19] American Heart Association Angina pectorishttp://www.americanheart.org/presenter.jhtml?identifier=4472. Accessed March 14, 2011

[B20] Dogan S Duivenvoorden R Grobbee DE Completeness of carotid intima media thickness measurements depends on body composition: the RADIANCE 1 and 2 trials J Atheroscler Thromb 2010 17 526 535 10.5551/jat.3269 20228610

[B21] Hodis HN Mack WJ LaBree L The role of carotid arterial intima-media thickness in predicting clinical coronary events Ann Int Med 1998 128 262 269 9471928 10.7326/0003-4819-128-4-199802150-00002

[B22] Rosenson RS Freeman MW Saperia GM Measurement of serum lipids and lipoproteinshttp://www.uptodate.com/contents/measurement-of-serum-lipids-and-lipoproteins

[B23] Caballero AE Metabolic and vascular abnormalities in subjects at risk for type 2 diabetes: the early start of a dangerous situation Arch Med Res 2005 36 241 249 10.1016/j.arcmed.2005.03.013 15925014

[B24] Galassi A Reynolds K He J Metabolic syndrome and risk of cardiovascular disease: a meta-analysis Am J Med 2006 119 812 819 10.1016/j.amjmed.2006.02.031 17000207

[B25] Hassinen M Komulainen P Lakka TA Metabolic syndrome and the progression of carotid intima-media thickness in elderly women Arch Intern Med 2006 166 444 449 16505265 10.1001/archinte.166.4.444

[B26] Isomaa B Almgren P Tuomi T Cardiovascular morbidity and mortality associated with the metabolic syndrome Diabetes Care 2001 24 683 689 10.2337/diacare.24.4.683 11315831

[B27] Tzou WS Douglas PS Srinivasan SR Increased subclinical atherosclerosis in young adults with metabolic syndrome: the Bogalusa Heart Study J Am Coll Cardiol 2005 46 457 463 10.1016/j.jacc.2005.04.046 16053958

[B28] World Health Organization Definition, Diagnosis and Classification of Diabetes Mellitus and its Complications. Part 1: Diagnosis and Classification of Diabetes Mellitus 1999 Geneva, Switzerland: World Health Organization

[B29] Laaksonen DE Lakka HM Niskanen LK Metabolic syndrome and development of diabetes mellitus: application and validation of recently suggested definitions of the metabolic syndrome in a prospective cohort study Am J Epidemiol 2002 156 1070 1077 10.1093/aje/kwf145 12446265

[B30] Meigs JB Wilson PW Nathan DM Prevalence and characteristics of the metabolic syndrome in the San Antonio Heart Framingham Offspring Studies Diabetes 2003 52 2160 2167 10.2337/diabetes.52.8.2160 12882936

[B31] Cartier A Côté M Lemieux I Sex differences in inflammatory markers: what is the contribution of visceral adiposity? Am J Clin Nutr 2009 89 1307 1314 10.3945/ajcn.2008.27030 19297456

[B32] Flanagan DE Vaile JC Petley GW Gender differences in the relationship between leptin, insulin resistance and the autonomic nervous system Regul Pept 2007 140 37 42 10.1016/j.regpep.2006.11.009 17187873

[B33] Kloting I kovacs P vanden Brandt J Sex-specific and sex-independent quantitative trait loci for facets of the metabolic syndrome in WOKW rats Biochem Biophys Res Commun 2001 284 150 156 10.1006/bbrc.2001.4932 11374884

[B34] Poulsen P Vaag A Kyvik K Genetic versus environmental etiology of the metabolic syndrome among male and female twins Diabetologia 2001 44 537 543 10.1007/s001250051659 11380071

[B35] Ahluwalia N Drouet L Ruidavets JB Metabolic syndrome is associated with markers of subclinical atherosclerosis in a French population-based sample Atherosclerosis 2006 186 345 353 10.1016/j.atherosclerosis.2005.07.021 16129441

[B36] O’Leary DH Polak JF Kronmal RA Cardiovascular Health Study Collaborative Research Group Carotid-artery intima and media thickness as a risk factor for myocardial infarction and stroke in older adults N Engl J Med 1999 340 14 22 10.1056/NEJM199901073400103 9878640

[B37] Davis PH Dawson JD Mahoney LT Increased carotid intimal-medial thickness and coronary calcification are related in young and middle-aged adults. The Muscatine study Circulation 1999 100 838 842 10.1161/01.CIR.100.8.838 10458720

[B38] Barrett-Connor E Sex differences in coronary heart disease. Why are women so superior? The 1995 Ancel Keys lecture Circulation 1997 95 252 264 10.1161/01.CIR.95.1.252 8994444

[B39] Cobble M Bale B Carotid intima-media thickness: knowledge and application to everyday practice Postgrad Med 2010 122 10 18 20107284 10.3810/pgm.2010.01.2091

[B40] Pannacciulli N De Pergola G Ciccone M Effect of family history of type 2 diabetes on the intima-media thickness of the common carotid artery in normal-weight, overweight, and obese glucose-tolerant young adults Diabetes Care 2003 26 1230 1234 10.2337/diacare.26.4.1230 12663602

[B41] Scuteri A Najjar SS Morrell CH The metabolic syndrome in older individuals: prevalence and prediction of cardiovascular events. The Cardiovascular Health Study Diabetes Care 2005 28 882 887 10.2337/diacare.28.4.882 15793190

[B42] Urbina EM Srinivasan SR Tang R Impact of multiple coronary risk factors on the intima-media thickness of different segments of carotid artery in healthy young adults (The Bogalusa Heart Study) Am J Cardiol 2002 90 953 958 10.1016/S0002-9149(02)02660-7 12398961

